# Critical Appraisal of Reimbursement List in Bosnia and Herzegovina

**DOI:** 10.3389/fphar.2017.00129

**Published:** 2017-03-17

**Authors:** Sabina Mujkic, Valentina Marinkovic

**Affiliations:** ^1^Regulatory Affairs Department, Alvogen Pharma d.o.o.Sarajevo, Bosnia and Herzegovina; ^2^Department of Social Pharmacy and Pharmaceutical Legislation, Faculty of Pharmacy, University of BelgradeBelgrade, Serbia

**Keywords:** health care system, legislation, reimbursement list, price differences, economic effects

## Pharmaceutical market of Bosnia and Herzegovina

One of the most challenging issues in health systems of middle-income countries is unequal access to medicines. When it comes to determining prices and reimbursement, different frames of price regulation and distribution margins, various methodological approaches, and tools for assessing the eligibility of costs for the insured are used (Jakovljevic et al., 2016a). Most countries in the European Union (EU) decided to regulate prices, at least when it comes to reimbursement lists (Rosian et al., 1998; Freemantle et al., 2001; Mrazek, 2002; Mossialos et al., 2004, 2006; Mossialos and Oliver, 2005; Vogler et al., 2005, 2008, 2009, 2011, 2015; Vogler and Habimana, 2014; Walley et al., 2005; Habl et al., 2006; Kazakov, 2007). Health legislation is one of the key elements in national and international activities related to health, as it plays a major role in development of a comprehensive support for individual and community health (Salihbasic, 2011).

The different and complex constitutional division as well as arrangement of price regulation in the healthcare system in BH causes huge losses. Because of lack of uniform legislation in this field and huge diversification in decision-making there is no adopted and unique methodology in price determination thus the key role in price determination in each entity have decision-makers involved in process.

The article's objective is to critically assess the methodological quality of decision-making process and Bosnian legislation for price determination as well as the reimbursement policy. Bosnian authority strives toward for regulation of prices according to external referent prices model, so this article also presents and outlines clear benefits that could be achieved if the method of price determination is homogenized between each entity according to referent prices especially in terms of price deviation outlined in this article.

Health system in Bosnia and Herzegovina (B&H) has fully divided jurisdiction between two entities and State District (Federation of Bosnia and Herzegovina, Republic of Srpska, and the Brcko District of B&H) further divided in entity Federation of B&H to 10 Cantons. This complex structure follows the administrative, constitutional frame of B&H which consist of two entities, State District and 10 Cantons in entity Federation of B&H. There is no single segment in this area, which would be a part of the jurisdiction of BH's state level authorities except for the BH's Law on Medicines and Medical Devices and the Agency for Medicines and Medical Devices^1^. Jurisdiction of the Agency for Medicines and Medical Devices at the state level is reflected in conducting the process of registration of medicines and medical devices.

In the Federation of B&H, the jurisdiction over the health sector is divided between the federal government and the Cantons, which means that the health sector is organized at the cantonal level and coordinated at the federal level (Salihbasic, 2011). As a part of the commitment to honor the citizen's rights to health care, it is essential that all countries ensure accessibility of essential medicines, i.e., medicines that meet the priority needs of the population (Hogerzeil, 2004, 2006; Hogerzeil et al., 2006). Reimbursement list in the Federation of BH (hereinafter: the Federal reimbursement list) contains only generic names. In addition to this, the cantonal positive lists of medicines are coordinated based on this list^2^, while the cantons are free in choosing trade names of a drug^1^. When creating a positive list of medicines in the cantons, the cantons must comply with the general and specific criteria for inclusion of drugs on the canton's positive lists of medicines. This list should contain information about a medicine as well as the mandatory acceptance of the prices established by the Federal Ministry of Health^3^. Medicine prices are established during negotiations of the Federal Ministry of Health with drug manufacturers. The price includes a manufacturing price of a drug, relevant customs duties, and other related costs of imports for imported drugs and the amount of wholesale margins (the maximum allowed margin is 8%)^4^.

In contrary to entity Federation of B&H, the health system in entity Republic of Srpska (RS) is fully centralized. Compulsory health insurance is regulated by the Health Insurance Fund of RS as an independent organization, while the control over the legality of the Fund is overseen by the ministry responsible for public health (Salihbasic, 2011). A reimbursement list in the Republic of Srpska is determined by the Health Insurance Fund. Afterwards, the entire determined list of drugs becomes available at the whole territory of RS^5^. In this entity, the Ministry of Health and Social Welfare and the Health Insurance Fund establish the system of pricing (Petrusic and Jakovljevic, 2015). The price of drugs stated on the list is formed based on the wholesale prices of medicines from the price list of the largest wholesalers, which includes VAT (internal pricing)^6^.

When it comes to Brcko District of B&H (BD), basic provisions of the entity's legal regulations are in place (Hogerzeil, 2004). The organizational structure of the health sector in BD was determined through forming of the Department of Health. This department is in charge of making a proposal of a reimbursement list containing generic names of drugs. It was modeled after the most favorable drug list in one of the entities, either the Federation B&H or the RS^6^. Prices for the list of medicines of BD are determined by the sum of the minimum manufacturer prices and margins, which the Government of BD set in the amount of 20%^7^.

## The data report methods

### Materials and methods

The study features critical analysis of drug prices for period 2011–2015, current methods of price determination, and impact on a budget. We performed a cross-sectional study using the official published data for the period 2011–2015.

The data from the official documents of Bosnia and Herzegovina relating to the price of drugs on reimbursement lists from certain regions in Bosnia and Herzegovina, spanning the period from 2011 to 2015, was compared. Each region has published different price list and it is available for each region, Brcko District,^6^^,^^7^^,^^8^, Federation BH^9^, and Republic of Srpska^10^.

In more recent times in Bosnia and Herzegovina, there is a legislative tendency to determine prices for medicines according to external reference pricing takes into account benchmark countries, namely: Serbia, Croatia, Slovenia, and Italy and Bulgaria, as substitute countries (Jakovljevic, 2013). In accordance with the current proposals of institutions in Bosnia and Herzegovina, this paper presents economic effects of the actual situation in Bosnia and Herzegovina, compared with the prices in Serbia (the prices of drugs on List A—Group A. Drugs that are prescribed and dispensed via a medical prescription, and List A1—drugs which are prescribed and dispensed via medical prescriptions, which have a therapeutic parallel with the medicines from List A, in the period from 2011 to 2015.)^11^. Each year, the exchange rate for the month of June is used for the stated period. The prices are calculated and presented in convertible marks (BAM)^12^.

The total number of different INN's listed on the reimbursement lists in the Federation BH and the RS is the same—138. This number is significantly lower in BD, where it only amounts to 75. What is interesting is that there is no representative on the BD reimbursement list within the two ATC groups (the D group—preparations for skin application and S group—medicines which affect the sensory organs). Average prices of medicines on lists of medicines in the Federation B&H, RS, and BD, show considerable variations, both regarding the average price, and the average price per individual ATC group. To be specific, the average price of drugs on the reimbursement list of the Federation B&H is 12.71 BAM. This price is much higher in RS and BD, as it amounts to 29.44 and 17.07 BAM, respectively. This difference is most striking when it comes to the average price of medicines from Group L. Although, the average price in this drug group in the Federation B&H was only 34.26 BAM, the RS reimbursement list of drugs shows that the medium price was as high as 139.64 BAM.

### Prices inequalities and impact on healthcare system

The consequences of price differences and differences in the structure of the reimbursement lists for individual regions have had the most impact on the treatment and quality of life of the patients and the entire healthcare system.

The data from the Federation BH presented in Table 1, show a decline in prices in accordance with the reference prices, but significant deviations are still present. Reduction of referent prices from 2011 to 2015 was clearly showed. Regardless gradual reduction of prices of every region, some INNs, such as valproic acid + sodium valproate, did not follow the trend of referent price reduction. In 2015 price deviation of valproic acid + sodium valproate in FBH was 54%, in RS was 72%, and in BD 72%.

**Table 1:** **Differences in prices for Reimbursement list for epileptic group of medicines in Bosnia and Herzegovina in comparison with reference prices for the period from 2011 to 2015**.

**ATC**	**INN**	**Form and pack size**	**Prices 2011**	**Ref. prices 2011**	**(%)^a^**	**Prices 2012**	**Ref. prices 2012**	**(%)^a^**	**Prices 2013**	**Ref. prices 2013**	**(%)^a^**	**Prices 2014**	**Ref. prices 2014**	**(%)^a^**	**Prices 2015**	**Ref. prices 2015**	**(%)^a^**
**FEDERATION BOSNIA AND HERZEGOVINA**																	
N03AX09	Lamotrigine	Tablet, 30 × 100 mg	25.5	23.1	9	25.5	16.32	36	22.1	18.29	17	17.87	17.65	1	16.08	9.29	42
N03AX11	Topiramate	Tablet, 60 × 100 mg	72	73.58	−2	72	44.88	38	72	49.03	32	52.58	44.42	16	47.32	30.06	36
N05AH02	Clozapine	Tablet, 50 × 100 mg	35.36	35.33	0	35.36	24.27	31	35.35	28.35	20	31.36	27.36	13	31.36	17.79	43
N03AA02	Phenobarbitone	Tablet, 30 × 100 mg	3.27	2.93	10	3.27	2.48	24	3.3	2.89	12	3.3	2.67	19	3.3	2.75	17
N03AE01	Clonazepam	Tablet, 30 × 2 mg	N/A	3.08	N/A	N/A	2.57	N/A	N/A	3.01	N/A	N/A	2.51	N/A	N/A	1.63	N/A
N03AF01	Carbamazepine	Tablet, 50 × 200 mg	6	3.88	35	6	3.23	46	13.7	4.52	67	10.28	4.34	58	10.28	4.27	58
N03AF02	Oxcarbazepine	Oral suspension	N/A	43.08	N/A	N/A	N/A	N/A	59.25	41.78	29	50.36	N/A	N/A	45.32	N/A	N/A
N03AG01	Sodium valproate	Tablet, 100 × 300 mg	34.52	N/A	N/A	34.52	N/A	N/A	N/A	N/A	N/A	N/A	N/A	N/A	24.15	N/A	N/A
N03AG02	Valproic acid, Sodium valproate	Film-coated tablet, 30 × 500 mg	N/A	9.47	N/A	N/A	7.89	N/A	17.3	8.86	49	12.11	5.59	54	12.11	5.61	54
N03AX12	Gabapentin	Capsule, 50 × 300 mg	26	19.48	25	26	16.07	38	24.1	18.62	23	22.17	17.43	21	19.95	12.58	37
N03AX14	Levetiracetam	Film-coated tablet, 60 × 500 mg	N/A	126.38	N/A	N/A	93.06	N/A	N/A	103.69	N/A	68.46	38.73	43	54.77	20.38	63
**THE REPUBLIC OF SRPSKA**																	
N03AX09	Lamotrigine	Tablet, 30 × 100 mg	56.83	23.1	59	56.83	16.32	71	30.41	18.29	40	30.41	17.65	42	29.82	9.29	69
N03AX11	Topiramate	Tablet, 60 × 100 mg	158.77	73.58	54	158.77	44.88	72	84.23	49.03	42	84.23	44.42	47	84.23	30.06	64
N05AH02	Clozapine	Tablet, 50 × 100 mg	44.44	35.33	20	44.44	24.27	45	41.37	28.35	31	41.37	27.36	34	41.37	17.79	57
N03AA02	Phenobarbitone	Tablet, 30 × 100 mg	3.63	2.93	19	3.63	2.48	32	3.63	2.89	20	3.63	2.67	26	3.63	2.75	24
N03AE01	Clonazepam	Tablet, 30 × 2 mg	6.26	3.08	51	6.26	2.57	59	N/A	3.01	N/A	N/A	2.51	N/A	6.26	1.63	74
N03AF01	Carbamazepine	Tablet, 50 × 200 mg	4.33	3.88	10	4.33	3.23	25	4.32	4.52	−5	4.32	4.34	0	4.32	4.27	1
N03AF02	Oxcarbazepine	Oral suspension	54.02	43.08	20	54.02	N/A	N/A	54.02	41.78	23	54.02	N/A	N/A	54.02	N/A	N/A
N03AG01	Sodium valproate	Tablet, 100 × 300 mg	N/A	N/A	N/A	N/A	N/A	N/A	N/A	N/A	N/A	N/A	N/A	N/A	20.23	N/A	N/A
N03AG02	Valproic acid, Sodium valproate	Film-coated tablet, 30 × 500 mg	20.83	9.47	55	20.83	7.89	62	20.83	8.86	57	20.83	5.59	73	20.23	5.61	72
N03AX12	Gabapentin	Capsule, 50 × 300 mg	28.31	19.48	31	28.31	16.07	43	28.31	18.62	34	28.31	17.43	38	28.15	12.58	55
N03AX14	Levetiracetam	Film-coated tablet, 60 × 500 mg	96.81	126.38	−31	96.81	93.06	4	96.81	103.69	−7	96.81	38.73	60	96.81	20.38	79
**BRCKO DISTRICT**																	
N03AX09	Lamotrigine	Tablet, 30 × 100 mg	N/A	23.1	N/A	36.65	16.32	55	29.85	18.29	39	20.9	17.65	16	20.9	9.29	56
N03AX11	Topiramate	Tablet, 60 × 100 mg	N/A	73.58	N/A	N/A	44.88	N/A	N/A	49.03	N/A	N/A	44.42	N/A	N/A	30.06	N/A
N05AH02	Clozapine	Tablet, 50 × 100 mg	N/A	35.33	N/A	44.44	24.27	45	41.35	28.35	31	36.7	27.36	25	36.7	17.79	52
N03AA02	Phenobarbitone	tablet, 30 × 100 mg	3.1	2.93	5	3.1	2.48	20	3.1	2.89	7	3.1	2.67	14	3.63	2.75	24
N03AE01	Clonazepam	Tablet, 30 × 2 mg	N/A	3.08	N/A	N/A	2.57	N/A	N/A	3.01	N/A	N/A	2.51	N/A	N/A	1.63	N/A
N03AF01	Carbamazepine	tablet, 50 × 200 mg	3.7	3.88	−5	3.7	3.23	13	3.7	4.52	−22	3.7	4.34	−17	4.32	4.27	1
N03AF02	Oxcarbazepine	Oral suspension	N/A	43.08	N/A	N/A	N/A	N/A	N/A	41.78	N/A	N/A	N/A	N/A	N/A	N/A	N/A
N03AG01	Sodium valproate	Tablet, 100 × 300 mg	34.53	N/A	N/A	34.53	N/A	N/A	34.53	N/A	N/A	28.25	N/A	N/A	28.25	N/A	N/A
N03AG02	Valproic acid, Sodium valproate	Film-coated tablet, 30 × 500 mg	17.8	9.47	47	17.8	7.89	56	17.8	8.86	50	17.8	5.59	69	20.23	5.61	72
N03AX12	Gabapentin	Capsule, 50 × 300 mg	N/A	19.48	N/A	N/A	16.07	N/A	N/A	18.62	N/A	N/A	17.43	N/A	N/A	12.58	N/A
N03AX14	Levetiracetam	Film-coated tablet, 60 × 500 mg	N/A	126.38	N/A	N/A	93.06	N/A	N/A	103.69	N/A	N/A	38.73	N/A	N/A	20.38	N/A

*N/A, not available*.

a *(%), prices deviation in percent*.

For example, the deviation in prices for lamotrigine in 2011 was 9% from the reference price, in 2012 36%, 2013 was 17%. 2014 witnessed a deviation of 1%, which is a subtle deviation from the reference price, however, in 2015, there was a sudden increase in the difference in prices, which amounted to 42%. This is rather significant given that this was the year of great market preparations and policy changes related to the new regulations on the method of price control, the method of forming prices of medicines and the manner of reporting on the prices of medicines in B&H. Also, the prices between entities are significantly different, and the higher prices were observed in the Republic of Srpska.

However, in RS, the deviations for the same medicine are even greater. For example, in 2011 was 59%, in 2012, 71%, in 2013, 40%, in 2014, 42%, and in 2015, 69%. It is worrying that according to reports of the Agency for Medicinal Products and Medical Devices, there was no reduction of total costs for the aforementioned drug considering reduction of prices^13^ (Supplementary Table 1).

In 2013, there was a sudden drop in the price of medicines in RS, but also a slight decline in other regions as well. That year, there was a reduction in the costs for the drug lamotrigine, however, what followed was a growth trend of total costs which should be worrisome. It is tough to establish adequate monitoring of reimbursement lists in BH due to lack of price control mechanisms, control of consumption, and drug prescriptions, as a result of decentralized regulations. It is also becoming increasingly difficult to monitor the drug supply within a payer rights.

The dataset has been submitted to a public repository Figshare, and it is available on https://figshare.com/s/07fa8d6d5a1e7a0eeaa4. Data has been uploaded as Excel file while the Appendix with all INNs (https://figshare.com/s/506f6aea67ab6208aaad) and the results are in PDF formats. Readers are free to access and reuse these publicly available data at the links provided above.

### Reimbursement and pricing policies

These results indicate that there are a high inequality and instability in the health sector. If we take into account the reference prices in the Republic of Serbia, as well as the mechanisms of rationalization of prescribing drugs, the result could be to reduce the cost of treatment, which would consequently lead to large savings in financial resources. These resources could further be allocated toward the prevention and stabilization of the health care system.

Significant expenses that occur due to the inability to control the prices, impede the progress of the health system and the introduction of new therapies, which are more efficient and cost-effectiveness.

Limitations of a decentralized health care system in BH, whether political, administrative, or fiscal, call into question financial sustainability of the health sector in BH^14^.

Inefficiency of the organizational model present in BH and limitations of institutional capacities and institutional fragmentation of every region, present the biggest problem of this concept of health care organization and causes an unequal access to health care (Jakovljevic and Ogura, 2016). However, this is not the case in developed countries with decentralized systems. In fact, this concept proved to be very convenient because it allowed access to medicines and other health care services to the marginalized population and population living in geographically remote areas (Forouzanfar et al., 2016). Lack of adequate homogeneous legislation within BH and apparent differences in prices, constitutional and administrative fragmentation, as well as health sector jurisdiction fragmentation contribute to enormous economic losses, which further weaken the overall health system in Bosnia and Herzegovina. One of the ways to approach solving of this problem should be centralization of the decision-making processes related to medications, clear guidelines, and policies on drugs as well as the laws and by-laws in BH, preferably through a better implementation of these regulations and policies in a decentralized system of supply, necessary complemented with in-depth constitutional, and administrative reform. Only this way full effect on health care system could be achieved.

Most of the EU countries have developed detailed criteria (e.g., cost-efficiency, relative efficiency, the need for medication), to help them decide when and how much is refundable. Health Economics also called the 'Fourth Hurdle' (Cohen et al., 2007; Forouzanfar et al., 2016) is something that is increasingly being used in the decision-making process (Drummond et al., 1997, 2010; Cohen et al., 2008). The pricing policy is most often determined institutionally, where drug prices are determined by legal regulations (e.g., legislation, regulations, and so forth). One such example should be implemented in the health care system in B&H with presented clear benefits of such system implementation.

## Conclusive remarks

The relationship between economic development and health care system are interrelated and dependent on one another. In most cases, the primary concern are financial costs of health care services and social security schemes. However, one major impact on all of the above are primarily the overall costs of illness and early death for the society and the individual. The concept of developing a health care system has many similarities with the economic development (Jakovljevic et al., 2016b). Both processes are the result of activities which involve many sectors of society, as well as the population as a whole, through individual and collective decisions and actions. Social deprivation, along with economic inequalities and housing conditions, results in a lower quality of life and shorter life expectancy (Jakovljevic et al., 2015). Medicines are a paramount segment of every health system, not only for treating a disease but also because of a high consumption of available resources in the health care system toward drugs. The analysis of the current situation shows that, most often, significant funds are spent on drugs that do not have adequate therapeutic value, and this is in addition to losses occurring as a result of a jurisdiction conflict and overlaps in all regions of the country. This means that the medicine sector, as well as the entire health care system in Bosnia and Herzegovina is in need of re-organization. One of the more important factors affecting the financial condition of our overall health care system is the present deviation in pricing and the inability to control them. Reimbursement regulations and reference pricing are the key mechanisms employed by government and regulatory bodies to manage pharmaceutical costs (Seget, 2003). Following new trends and introducing new methods of determining the efficiency of drugs in relation to the resources expended, the health care services in B&H can be improved to a great extent which would clearly present biggest benefit to end users of healthcare system of Bosnia and Herzegovina.

## Author contributions

All authors listed, have made a substantial, direct and intellectual contribution to the work, and approved it for publication.

### Conflict of interest statement

The authors declare that the research was conducted in the absence of any commercial or financial relationships that could be construed as a potential conflict of interest.

## Footnotes

^1^Medicinal Product Act. “Official Gazette of Federation” BH No. 51/10 and 29/05.

^2^Health Care Act. “Official Gazette of Federation” BH No. 30/97, with amendments Nos. 7/02 and 70/08.

^3^The decision of Essential list of medicines. “Official Gazette of Federation BH” No. 75/11; The decision of List of medicines, Solidarity Fund of Federation BH. “Official Gazette of Federation BH” No. 67/11.

^4^The decision on the criteria for inclusion of medicinal products on the list of medicines on prescription reimbursed from Health Insurance Fund of the Republic of Srpska. “Official Gazette of Republic of Srpska” No. 2/10.

^5^Ministry of Health and Social Care, The strategy in the field of medicine by 2012, 16.

^6^Health Insurance Act, Brcko District BH, “Official Gazette of Brcko District” BH No. 1/02, 7/02, 2/8, 19/7, and 34/08.

^7^The decision on the methodology of determining the reference price of medicine from List of essential medicines for the needs of insured persons, the Health Insurance Fund of Brcko District BH, “Official Gazette Brcko District BH,” No. 52/11.

^8^The decision on establishing the list of essential medicines for the needs of insured persons the Health Insurance Fund of Brcko District, 04.04.2013; Table with established changes in prices of medicines with the list of essential medicines for the needs of insured persons of Health Insurance Fund of Brcko District B&H, 2014; Established prices of essential medicines for the needs of insured persons the Health Insurance Fund of Brcko District, Health Insurance Fund, 07.05.2015.

^9^The decision for the list of essential drugs required to provide health care within the standards of the mandatory health insurance in Federation BH, “Official Gazette of Federation” BH, Nos. 75/11, 56/13, 74/14, and 94/15.

^10^Pricelist for List of medicine from A, A1, B list. Health Insurance Fund of Republic of Srpska, 21.10.2008, 01.10.2013, and 15.03.2015.

^11^Ordinance of List of medicines prescribed and issued at the expense of mandatory health insurance, “Official Gazette of the Republic of Serbia“ No. 28/11.; Ordinance of List of medicines prescribed and issued at the expense of mandatory health insurance, “Official Gazette of Republic of Serbia,” dated 20.03.2012. Prices in Serbia in 2013. Available: http://media.dzindjija.rs/2013/09/Lista-lekova-Sl-glasnik-76_13.pdf (accessed: 21.06.2013). Ordinance of List of medicines prescribed and issued at the expense of mandatory health insurance, “Official Gazette of Republic of Serbia,” No. 7/14. Ordinance of List of medicines prescribed and issued at the expense of mandatory health insurance, “Official Gazette of Republic of Serbia,” No. 7/14.

^12^Exchange rate, dated 01.06.2011.: https://www.unicredit.ba/ba/stanovnistvo/tecajna_lista.html; Exchange rates, dated 01.06. 2012: https://www.unicredit.ba/ba/stanovnistvo/tecajna_lista.html; Exchange rate, 03.06.2013: https://www.unicredit.ba/ba/stanovnistvo/tecajna_lista.html; Exchange rate, dated 02.06.2014: https://www.unicredit.ba/ba/stanovnistvo/tecajna_lista.html; Exchange rate, dated 01.06.2015.: https://www.unicredit.ba/ba/stanovnistvo/tecajna_lista.html (accessed: 06.12.2016).

^13^The report of distribution of medicines licensed for BH market for period from 2011 to 2015. The Agency for medicinal products and Medical Devices, Banja Luka, 2012, 2013, 2014, 2015, 2016.

^14^Strategic Plan and the development of health in Federation of Bosnia and Herzegovina for period 2008 till 2018, Ministry of Health Federation of Bosnia and Herzegovina, Sarajevo 2008. Available: http://www.fmoh.gov.ba/images/federalno_ministarstvo_zdravstva/zakoni_i_strategije/strategije_i_politike/dokumenti/usvojeni_strateski_plan_1_.pdf
